# Increased NLRP1 mRNA and Protein Expression Suggests Inflammasome Activation in the Dorsolateral Prefrontal and Medial Orbitofrontal Cortex in Schizophrenia

**DOI:** 10.3390/biom14030302

**Published:** 2024-03-04

**Authors:** Ena Španić Popovački, Dora Vogrinc, Heidi R. Fuller, Lea Langer Horvat, Davor Mayer, Janja Kopić, Klara Pintarić, Mirjana Babić Leko, Mihaela Pravica, Željka Krsnik, Darko Marčinko, Marina Šagud, Patrick R. Hof, Mihovil Mladinov, Goran Šimić

**Affiliations:** 1 Department of Neuroscience, Croatian Institute for Brain Research, University of Zagreb School of Medicine, 10000 Zagreb, Croatiallanger@hiim.hr (L.L.H.);; 2 Wolfson Centre for Inherited Neuromuscular Disease, RJAH Orthopaedic Hospital, Oswestry SY10 7AG, UK; h.r.fuller@keele.ac.uk; 3 School of Pharmacy and Bioengineering, Keele University, Keele ST5 5BG, UK; 4 Department of Forensic Medicine, University of Zagreb School of Medicine, 10000 Zagreb, Croatia; 5 Department of Psychiatry and Psychological Medicine, University Hospital Center Zagreb, 10000 Zagreb, Croatia; predstojnik.psi@kbc-zagreb.hr (D.M.);; 6 University of Zagreb School of Medicine, 10000 Zagreb, Croatia; 7 Nash Family Department of Neuroscience, Center for Discovery and Innovation, and Friedman Brain Institute, Icahn School of Medicine at Mount Sinai, New York, NY 10019, USA; patrick.hof@mssm.edu; 8 Department of Psychiatry and Psychotherapy, University of Rostock, Gehlsheimer Str. 20, 18147 Rostock, Germany

**Keywords:** schizophrenia, NLRP1 inflammasome, predictive processing, prefrontal cortex, pyramidal neurons

## Abstract

Schizophrenia is a complex mental condition, with key symptoms marked for diagnosis including delusions, hallucinations, disorganized thinking, reduced emotional expression, and social dysfunction. In the context of major developmental hypotheses of schizophrenia, notably those concerning maternal immune activation and neuroinflammation, we studied *NLRP1* expression and content in the postmortem brain tissue of 10 schizophrenia and 10 control subjects. In the medial orbitofrontal cortex (Brodmann’s area 11/12) and dorsolateral prefrontal cortex (area 46) from both hemispheres of six schizophrenia subjects, the *NLRP1* mRNA expression was significantly higher than in six control brains (*p* < 0.05). As the expression difference was highest for the medial orbitofrontal cortex in the right hemisphere, we assessed NLRP1-immunoreactive pyramidal neurons in layers III, V, and VI in the medial orbitofrontal cortex in the right hemisphere of seven schizophrenia and five control brains. Compared to controls, we quantified a significantly higher number of NLRP1-positive pyramidal neurons in the schizophrenia brains (*p* < 0.01), suggesting NLRP1 inflammasome activation in schizophrenia subjects. Layer III pyramidal neuron dysfunction aligns with working memory deficits, while impairments of pyramidal neurons in layers V and VI likely disrupt predictive processing. We propose NLRP1 inflammasome as a potential biomarker and therapeutic target in schizophrenia.

## 1. Introduction

Schizophrenia (SZ) is a persistent, multifactorial mental condition that affects the generation of thoughts, reality perception, cognitive, linguistic, and emotional experience and expression, as well as social relationships [1]. It is characterized by delusions and hallucinations of variable severity (positive symptoms not normally seen in people who do not have SZ) and a disorganized flow of thoughts with consequent incoherent speech. According to the *Diagnostic and Statistical Manual of Mental Disorders*, Fifth Edition, Text Revision (DSM-5-TR), the diagnosis of SZ requires the presence of two of these three symptoms for a significant portion of time during one month (or less if successfully treated) [2].

The Eleventh Edition of the *International Classification of Diseases* (ICD-11) by the World Health Organization [3] has harmonized its criteria to better align with the DSM’s criteria. ICD-11 suggests categorizing symptoms into six groups (positive, negative, depressive, manic, psychomotor, and cognitive), requiring the presence of at least two symptoms during a month. Among these two, one must be from the group of so-called basic symptoms (delusions, hallucinations, thought disorder, and distorted perception of reality) [3], facilitating easier comparisons and enhancing clinical usability [4]. Different assessment tools, such as the Clinician-Rated Dimensions of Psychosis Symptom Severity (C-RDPSS) introduced in the DSM-5, are designed to evaluate various groups of symptoms on a scale from 0 (absence of the symptom) to 5 (presence of the symptom in a severe form or to a significant extent), as reported by the individual in the previous seven days, and to monitor treatment success. The main challenge in diagnosing and treating SZ lies in its reliance on symptoms of a psychological nature rather than on clearly delineated pathophysiological or neurobiological mechanisms [5]. The current diagnostic criteria for SZ remain dichotomous, determining the presence or absence of the illness, and do not facilitate early diagnosis; instead, they require the progression of symptomatology before diagnosis. Consequently, there is a crucial need to align these criteria with contemporary scientific knowledge and refine them based on clinical neuroscience, genetic testing, biomarkers, neuroimaging, and personalized pharmacotherapy [6].

SZ is characterized as a disconnection syndrome that arises from the aberrant maturation and connectivity of the prefrontal cortex (PFC). This condition elevates the threshold for conscious perception due to the aberrant synaptic plasticity of glutamate, particularly the hypofunction of *N*-methyl-D-aspartate receptors (NMDARs), which occurs earlier during or before the juvenile period. This stage is later in adolescence influenced by the dysfunction of various neurotransmitter systems, mainly dopamine, serotonin, acetylcholine, and GABA (for a review, see [7]). Neuroinflammation is increasingly considered a key mechanism involved in this transition. While neuroinflammation plays a key role in the pathogenesis of many brain diseases, including Alzheimer’s disease [8,9,10], Huntington’s disease, Parkinson’s disease, drug use disorders, depression, and anxiety disorders, the relationship between the NLRP inflammasome and schizophrenia, autism, and many other neuropsychiatric disorders remains unknown (for a review, see [11]).

The NMDAR hypofunction hypothesis of SZ is derived from the observation that specific non-competitive NMDAR antagonists, such as phencyclidine and ketamine, elicit behaviors reminiscent of all three types of SZ symptoms (positive, negative, and cognitive) in humans [12,13]. It is posited that this hypofunction is responsible for cognitive and social deficits. This viewpoint is reinforced by the limited efficacy of antipsychotic drugs targeting the dopaminergic system in addressing these deficits in SZ and the development of treatment-resistant SZ (TRS) affecting up to a third of individuals with SZ [14,15]. The consequences of these alterations include deficits in both local circuitry processing within the PFC and its long-range connectivity, causing impairments in predictive processing—the key pathophysiological disturbance in SZ [16,17]. 

When normal predictive coding, mediated by layer V PFC top-down (feedback) projections to the visual area V4, is experimentally suppressed in monkeys during a task-altering visual predictability task, this intervention results in a lack of the normal increase in α and β electroencephalography (EEG) power that conveys expected inputs (power modulation is stimulus-specific). Simultaneously, unpredicted stimuli do not elicit a rise in spiking and γ band activity, as observed during normal conditions [18]. Similarly, it has been demonstrated that a greater prior expectation of volatility is elevated in individuals with higher paranoia (i.e., the belief that others intend harm) and is associated with persecutory delusion severity in SZ patients—the most common delusions in SZ, representing the outermost edge of the paranoia continuum [19]. Predictive processing not only encompasses sensory systems but is also crucial in approximating the consequences of impending motion [20], language [21,22], and other higher-order functions of the brain. Most of the frontal lobe areas likely serve this function of predictive coding in the motor system, a process termed “active inference” [23]. Probably the only exception to this rule is Brodmann’s area 4 (M1, the primary motor cortex), which does not receive prediction error information and is thus agranular in adults (lacks a well-developed layer IV). The likely underlying reason for this is the postnatal recession of layer IV due to the neurodevelopmental acquisition of motor skills and the fact that prediction errors are efficiently handled at the periphery through the spinal reflex arcs [24] and cerebellum.

Thalamocortical projections and corticothalamocortical loops also play a crucial role in downstream predictive processing, as substantiated by clinical neuroimaging research. For instance, in a study comparing resting-state functional magnetic resonance imaging (rs-fMRI) data from 90 SZ patients with controls, bilaterally excessive functional connectivity between the thalamus and sensory and motor areas of the cerebral cortex was identified in SZ. This excessive connectivity level correlated with the severity of the clinical picture and reduced the connectivity of the thalamus with the PFC, striatum, and cerebellum [25].

Comparably to the diagnostic process, therapy primarily targets individual symptoms of SZ rather than the underlying pathophysiological causes. Consequently, treatment relies on a trial-and-error approach. The predominant use of antipsychotics as the primary intervention to alleviate positive symptoms—delusions and hallucinations—often proves inadequate for many patients, especially considering their long-term effects [26]. Moreover, these medications exhibit limited efficacy against negative symptoms, such as social withdrawal and reduced motivation, as well as cognitive impairments like working memory and goal-directed behavior deficits attributed to the dysfunction of the dorsolateral PFC (DLPFC). Additionally, antipsychotics commonly lead to significant adverse effects, including metabolic, neurological (e.g., subtle but significant gray and white matter loss [27]), and cardiovascular issues and complications, affecting long-term adherence to treatment (for a comprehensive contemplation, see [26]).

Many paradigms of SZ pathogenesis and various causative and risk factors (environmental, psychosocial, developmental, genetic, epigenetic, and drug use disorders) converge to either hyperdopaminergic states in mesocortical projections from the ventral tegmental area to the ventral striatum (including nucleus accumbens) and PFC or neuroinflammatory changes. Hyperdopaminergic states, i.e., uncontrolled increases in presynaptic dopamine levels released without appropriate stimulation, are considered as a basis for positive symptoms of the disease [28]. They lead to misattributing importance to neutral sensory stimuli (aberrant salience), and thus individuals with SZ require longer periods to pay attention to and process irrelevant sensory stimuli compared to control subjects (Simon’s effect) [29,30]. Additionally, patients with SZ have notably decreased structural connectivity between the amygdala and MOFC via the uncinate fasciculus, whereas their increased amygdala activity may have a role in distress and the perception of threat related to auditory hallucinations; individuals with SZ also exhibit altered reward prediction and associated striatal and PFC activation, impaired reward learning, and impaired reward-modulated action selection (for a review, see [31]).

Neuroinflammatory changes play a significant role in SZ (for a review, see [32]). For instance, an increased numerical density of microglial cells in the frontal and temporal lobes has been reported in the brains of individuals with chronic SZ [33]. In animal models, chronic restraint, social isolation, and repeated social defeat lead to elevated microglial activity in the PFC and hippocampus. Calcia and collaborators proposed a two-hit hypothesis, suggesting that chronic and sustained microglial stimulation during prenatal/early life, due to interruptions/changes in the brain’s environment, induces an exaggerated microglial response later on. This primes microglial cells to be more sensitive to minor stimuli such as psychosocial stressors during adolescence/early adulthood [34]; see also [35,36]. Additionally, the humoral activation of brain microglia by patrolling monocytes is believed to affect stress-associated brain regions and amplify pro-inflammatory responses through interoceptive humoral pathways involving vascular endothelial IL-1 receptor type-1 signaling [37,38]. In comparison to healthy controls, a recent report indicates significantly elevated *NLRP3*, *P2RX7*, *IL-1β*, and *IL-18* gene expression levels in peripheral blood mononuclear cells of SZ patients, implicating systemic inflammatory changes in SZ [39].

Inflammasomes are protein complexes that assemble in the cytosol after the activation of the cytoplasmic nucleotide-binding oligomerization domain (NOD) of the NOD-like leucine-rich repeat-containing receptors (NLRs) in response to various damage-associated signals, pathogens, harmful substances, and metabolic perturbations [8]. The NLR family pyrin domain-containing 1 protein (NLRP1) is a 1429-amino-acid-long protein known to form an inflammasome complex and activate caspase-1 upon degradation of its N-terminal part by the proteasome in neurons. On the other hand, NLRP3 (NLR family pyrin domain-containing 3 protein) is the main NLRP family member in the brain, predominantly expressed in microglia. Thus far, it has been proposed that the activation of the NLRP3 inflammasome might be relevant to the pathogenesis of SZ [39,40,41,42]. A recent in vitro study on human induced pluripotent stem cells has shown a higher activation of the inflammasome in microglia of the SZ patients, which also impacted neuronal functions and led to a higher loss of the synapses [43]. 

NLRP3 activation in SZ is observed at the periphery [39,42] and can be triggered by various signals, including minute cholesterol crystals in early atherosclerotic lesions [44]. While the extent to which cholesterol crystals form in vivo remains a topic of ongoing investigations, one study has already shown that exposing bone marrow-derived macrophages to cholesterol-rich myelin debris is sufficient to engage NLRP3 inflammasome [45]. Possibly as a result of failed maturation, imaging and postmortem studies have revealed disturbed myelination and oligodendroglia-related processes in patients with SZ, including irregular gene expression and a reduced number of oligodendrocytes in the DLPFC [46]. This finding is of great potential importance as it links oligodendrocytes and myelin pathology in SZ to the activation of the inflammasome in the myeloid lineage, from which microglial cells are also derived. In line with maternal immune activation (MIA) and the activation of inflammasomes, a substantial number of studies have shown a more robust inflammatory response in patients with SZ (e.g., [47], which is also associated with worse clinical outcomes [48]).

Interestingly, while the NLRP3 inflammasome is dominantly expressed in microglia [8,49], a different type of inflammasome, NLRP1, is mostly expressed in the neurons of the central nervous system [50]. Its involvement in Alzheimer’s disease [9,51], cerebral ischemic [52] and reperfusion-ischemic injury [53], mesial temporal lobe epilepsy [54], and multiple sclerosis [55] has been documented earlier. This study aims to compare the expression of the NLRP1 inflammasome in the neurons of healthy control brains and SZ patients to assess whether the NLRP1 inflammasome plays a role in disease pathogenesis. The comparison of housekeeping gene expression offers additional insight into the underlying biological distinctions between SZ and control brains, contributing to a deeper understanding of the molecular basis of SZ.

## 2. Materials and Methods

### 2.1. Human Brain Tissue Samples

For this study, we analyzed brain samples of ten subjects with schizophrenia (SZ; six females and four males, mean age 55.9 ± 10.5 years) and ten control (CON) subjects (three females and seven males, mean age 57.8 ± 10.4 years). All SZ patients met the criteria for a diagnosis of SZ based on the *Diagnostic and Statistical Manual of Mental Disorders*, Fourth Edition, Text Revision [56], and, until their death, were under long-term treatment with either clozapine, olanzapine, or risperidone, with regular clinical follow-ups by at least one experienced psychiatrist. The symptoms described in the medical records of subjects with SZ whose brain samples were analyzed in our study were quite similar and included delusions, hallucinations, disorganized speech and behavior, as well as negative symptoms such as affective flattening, alogia, and avolition, all lasting for a period beyond 6 months from disease onset. Both SZ and CON brains were carefully selected to ensure that samples for analysis were not taken from subjects with a prior history of head trauma or other major neuropsychiatric disorders. CON samples were obtained from individuals comparable in age to the SZ samples, with no psychiatric or neurological illness. They were chosen to match the SZ group in terms of age and postmortem delay. Demographic data for both SZ and CON subjects are provided in Table 1.

**Figure 1:**
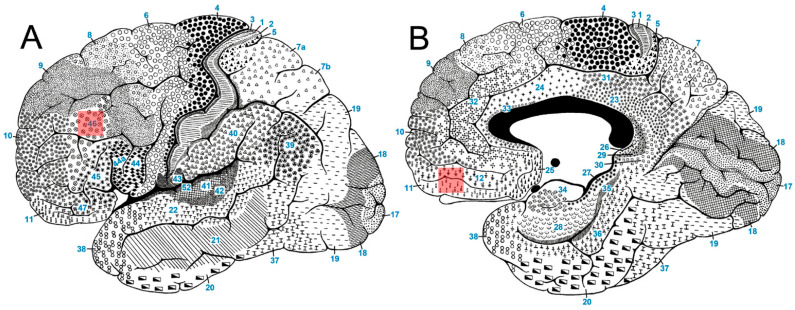
Locations of the analyzed samples. Blocks of brain tissue were taken from two different areas: (**A**) the dorsolateral prefrontal cortex (DLPFC, Brodmann’s area 46), and (**B**) the medial orbitofrontal cortex (MOFC, located between Brodmann’s areas 11 and 12). The specific locations from which these tissue blocks were taken are indicated by transparent red rectangles. The template for the figure is taken from our previous publications on Brodmann’s areas [63] and gene expression profiling in SZ [64].

The sampling of brain tissue from routine autopsies was conducted with the approval of the Central Ethical Committee of the University of Zagreb Medical School (Case no. 380-59-10106-23-111/93, Class: 641-01/23-02/01, from 11 December 2015). The postmortem samples included the orbitofrontal Brodmann’s areas 11/12 of the medial orbitofrontal cortex (MOFC) and the mid-frontal Brodmann’s area 46 of the dorsolateral prefrontal cortex (DLPFC) from both hemispheres (Figure 1). These samples were selected from the Zagreb Brain Bank Collection at the Croatian Institute for Brain Research in Zagreb, Croatia [57,58,59]. Each dissected sample had an approximate volume of around 0.5 cm^3^. The caudal segment of each block underwent staining with cresyl violet (Nissl stain). This staining process was instrumental in verifying the cytoarchitectural features within the analyzed Brodmann’s areas. Area 46 is demarcated dorsally by the granular frontal area 9, extending rostroventrally to the frontopolar area 10, and caudally connecting with the triangular area 45 [60]. On the other hand, area 11 is bordered rostrally and laterally by the frontopolar area 10, the orbital area 47, and the triangular area 45. Its caudal aspect is contiguous with the subgenual area 25. On the medial surface, it extends into the rostral area 12 [61,62,63].

### 2.2. NLRP1 Expression Analysis Using Microarray Procedure

After dissection, samples were immediately stabilized in RNAlater solution (Thermo Fischer Scientific, Waltham, MA, USA) and stored at −80 °C for subsequent analysis, as previously described [64]. Each microarray utilized 20–30 mg of brain tissue from BA46 or BA11/12. RNA isolation followed the protocol using the RNeasy Plus Mini kit from Qiagen (Venlo, The Netherlands). RNA concentration and quality were assessed using Agilent’s Bioanalyzer 2100 and RNA 6000 Nanochip Kit (both from Agilent Technologies, Santa Clara, CA, USA). RNA degradation was evaluated by 28S/18S ribosomal band peak ratios within the acceptable range of 1.5 to 2.0. The RNA integrity number (RIN) of each sample is given in Table 2. Total RNA underwent reverse transcription and was hybridized onto the Affymetrix HG-U133 Plus 2.0 array, employing the same protocol and gene identification names as the GeneChip Human Exon 1.0ST Arrays (Affymetrix, Santa Clara, CA, USA). This array examines over a million exons, spanning 17,868 NCBI Reference Sequence (RefSeq) transcripts. The procedure involved initial ribosomal RNA removal (RiboMinus Human/Mouse Transcriptome Isolation Kit, Invitrogen-Thermo Fischer Scientific, Waltham, MA, USA) to reduce background noise. Subsequently, double-stranded cDNA was amplified to antisense cRNA, purified, reverse-transcribed, fragmented, labeled, and hybridized into arrays. Gene expression values from microarray data (.cel files) were analyzed using Partek Genomic Suite software ver. 6.5 (Partek Incorporated, St. Louis, MO, USA). Data preparation included background noise correction, log2 transformation, quantile normalization, averaging probe signals by mean, filtering for a fold change of less than 1.5, and a *p*-value under 0.05 [65]. The entirety of the microarray transcriptome data are collected in our prior study [64]. As they contain all transcripts, including mRNA of the *NLRP1* gene listed in the dataset line 18,408 (of the Affymetrix EXON HuEx-1.0-st-v2 chip), we are also attaching them here as Supplementary Table S1 for reference. To find the ID of a gene of interest, one should utilize the conversion tool of the Database for Annotation, Visualization, and Integrated Discovery (DAVID, https://david.ncifcrf.gov/, accessed on 22 June 2023) [66]. The mRNA of the *NLRP1* gene corresponds to Entrez Gene ID 3742783 (*Homo sapiens*, NLR family pyrin domain containing 1, NCBI Reference Sequence: NM_033004.4). The values in dataset line 18,408, representing log2-transformed *NLRP1* mRNA signal intensities across all 48 samples, can be transformed back to expression levels of the *NLRP1* gene using the reverse procedure from logarithmic to exponential. For instance, a signal intensity of 3.55373 translates to an expression level of 2^3.55373^, which in turn is 11.74301, and so forth (see Table 2 for *NLRP1* expression levels across all samples).

### 2.3. Immunohistochemical Staining

Brain tissue samples were dissected from the MOFC, embedded in paraffin, and cut into 12 μm-thin slices for further immunohistochemical staining. Tissue sections were deparaffinized in xylene and rehydrated in the decreasing concentrations of ethanol (100%–twice, 96%, and 70%). Antigen retrieval was performed in a boiling citrate buffer (anhydrous citric acid solution 10 mM, pH 6), five times shortly (around 1 min) at high microwave power (700 W) and 20 min at low microwave power (300 W). Endogenous peroxidase activity was inhibited by incubating slides in 0.02% H_2_O_2_ in methanol (150 mL methanol and 50 mL water) for 30 min. Unspecific signals were blocked with 5% bovine serum albumin (BSA) + 0.5% Triton/PBS for 1 h at RT. The primary antibody (NLRP1, Abcam, Cambridge, UK, AB_776633) was diluted in blocking solution to working concentration (NLRP1 1:100). After overnight incubation with the primary antibody in a humidified chamber at 4 °C, slides were incubated with the goat antirabbit biotinylated secondary antibody (1:200) for 60 min (Vector Laboratories, Newark, CA, USA, AB_2336810, AB_2336811), followed by the application of the ABC complex also for 60 min at RT (Vector Laboratories, Newark, CA, USA, AB_2336810, AB_2336811). 3,3′-diaminobenzidine (Sigma-Aldrich, St. Louis, MO, USA, cat. #D0426) was used as a chromogen for developing peroxidase activity. Negative-control sections were not incubated in the primary antibodies. Sections were dehydrated before mounting in Histomount (Poly-Mount, Polysciences, Warrington, PA, USA, Cat. #08381-120). 

### 2.4. Quantification of NLRP-1-Immunoreactive Pyramidal Neurons

Stained sections were scanned by the Hamamatsu Nanozoomer 2.0. RS in 0.45 µm × 0.45 µm resolution (Hamamatsu Photonics K.K., Hamamatsu City, Japan) and analyzed in NDP.view2 program for digital visualization. Each NLRP1-positive pyramidal neuron was counted as one, and the results were presented as the number of immunoreactive cells per cortical area (in square millimeters) of interest (from the top of layer I to the bottom of layer VI). Cortical layers I-VI were identified based on the size, shape, and distribution of pyramidal cells, and the laminar attribution of labeled cells was determined by measuring the thickness of layers on adjacent Nissl-stained sections. Quantification was performed by D.V., who was blind to the group and the identity of the cases. Section analysis and images were obtained with an Olympus BX53 microscope (Olympus, Tokyo, Japan). The total number of all NLRP1-immunoreactive pyramidal neurons was determined in a selected section with the best staining, assessed using Image J software (National Institute of Health, Bethesda, MD, USA, https://imagej.nih.gov/ij/, accessed on 22 June 2023). The results were presented as the average density of NLRP1-immunoreactive neurons per analyzed area.

### 2.5. Statistical Analysis

Comparative analysis between SZ and CON groups regarding *NLRP1* transcript expression involved employing Student’s *t*-test. A *p*-value below the pre-specified α level of 0.05 indicated a statistical difference from the null hypothetical value (*p* < 0.05). To specifically evaluate if a given dataset followed a normal distribution, we tested using the Shapiro–Wilk test, as it is considered the most powerful one for small-sized samples. Due to a small sample size, differences in the number of NLRP1-positive pyramidal cells were analyzed with an approximation of normal distribution by both a parametric two-sample, two-tailed *t*-test and a non-parametric two-sample, two-tailed Mann–Whitney U-test (Wilcoxon rank-sum test). All statistical tests and graphs were made in GraphPad Prism version 9.3.1. (GraphPad Software, San Diego, CA, USA).

## 3. Results

### 3.1. Expression of NLRP1 mRNA in the DLPFC and MOFC

The results of *NLRP1* mRNA expression in the DLPFC and MOFC are summarized in Table 2. Due to a relatively small sample size, we conducted both a parametric *t*-test and a non-parametric Mann–Whitney test. The results indicated that in the DLPFC and MOFC from both hemispheres of six SZ subjects, the *NLRP1* mRNA expression was significantly higher than in six control brains (*t*-test and Mann–Whitney test *p*-values were lower than 0.05).

### 3.2. Immunohistochemical Analysis of the NLRP1 Protein Distribution in the MOFC

**Figure 2:**
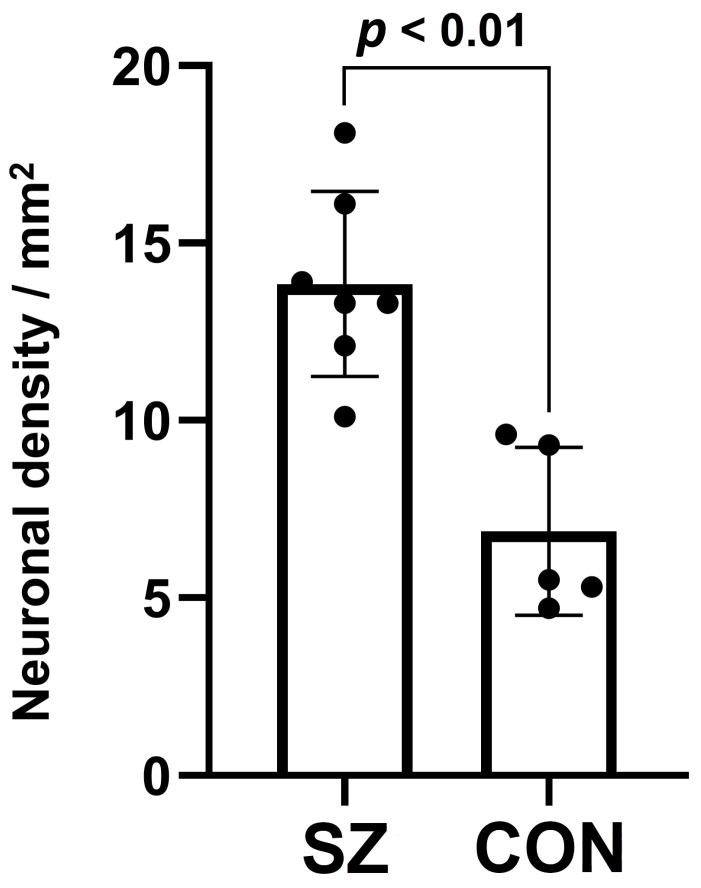
Graphical representation of the results from Table 3, showing that the average neuronal density of NLRP1-expressing MOFC pyramidal neurons in the RH is significantly higher (*p* < 0.01) in the group of seven schizophrenia (SZ) samples compared to the group of five control (CON) samples. MOFC, medial orbitofrontal cortex; RH, right hemisphere.

Considering that the most substantial mean difference in *NLRP1* mRNA expression between control and SZ subjects was found in tissue blocks from the MOFC of the right hemisphere (15.53 in SZ vs. 12.99 in controls, see Table 2), we proceeded with an immunohistochemical analysis on these specific blocks. We chose the best stained, representative MOFC section from each of the 12 blocks (5 from controls and 7 from SZ subjects) and quantified the number of NLRP1-positive pyramidal neurons in layers III, V, and VI. The results of the quantification are presented in Table 3 and Figure 2. They reveal that, in the MOFC of the right hemisphere of seven SZ subjects, NLRP1 protein immunoreactivity was detected in a significantly greater number of pyramidal neurons than in five control brains (both *t*-test and Mann–Whitney test *p*-values were lower than 0.01).

**Figure 3:**
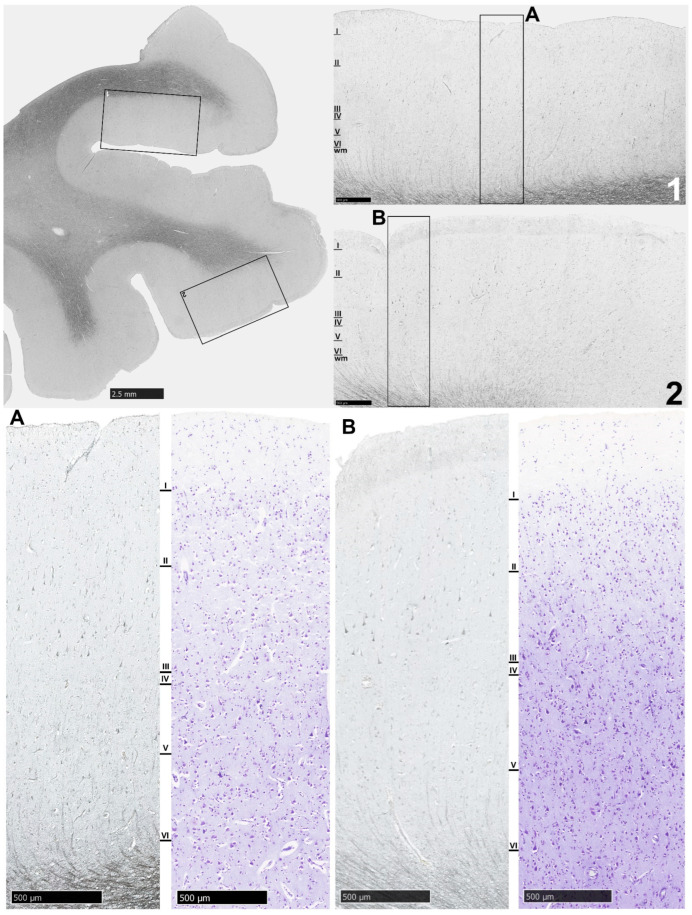
Microphotograph of NLRP1 immunocytochemical staining of MOFC from the right hemisphere of a selected control sample (CON9). A few pyramidal neurons in layer III are NLRP1-immunoreactive, while layers V and VI are almost devoid of labeled neurons. Insets (**A**) and (**B**) from (**1**) and (**2**), respectively, are Nissl-stained adjacent sections that allow cytoarchitecture to be appreciated. Note the thin layer IV. Scale bar = 2.5 mm. Scale bars in the insets (**1**,**2**) as well as (**A**,**B**) = 500 μm.

**Figure 4:**
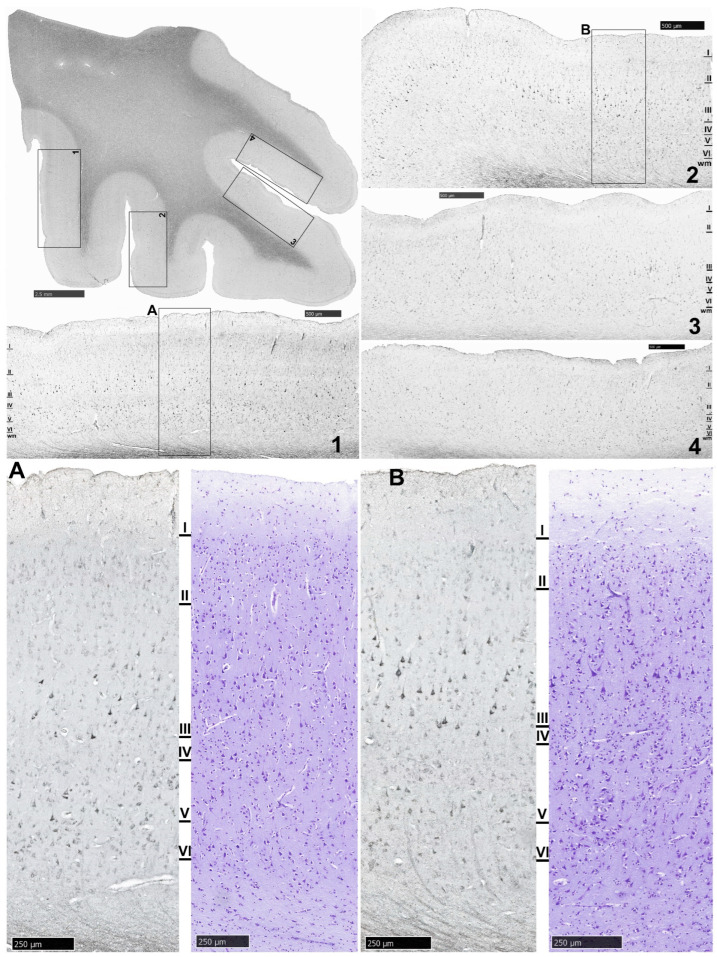
Microphotographs of NLRP1 immunocytochemical staining of MOFC from the right hemisphere of an SZ sample (SZ6). Many pyramidal neurons of layer III as well as pyramidal neurons in layers V and VI are NLRP1-immunoreactive. Layer IV is virtually free from NLRP1-immunoreactive cells. Also, note cortical atrophy and the paucity of the white matter relative to the 9-year older control sample shown in Figure 3. Insets (**A**) and (**B**) from (**1**) and (**2**), respectively, are Nissl-stained adjacent sections that allow cytoarchitecture to be appreciated. Scale bar = 2.5 mm. Scale bars in the insets (**1**–**4**) = 500 μm. Scale bars in (**A**,**B**) = 250 μm.

NLRP1 protein expression in the MOFC of one selected control sample (CON9) is shown in Figure 3. For comparison, a selected sample of NLRP1 protein expression in the MOFC of one SZ case is presented in Figure 4.

### 3.3. Expression of Housekeeping Genes

There is no ideal reference housekeeping gene consistently expressed in all experimental conditions, regardless of the tissue type and disease state [67]. Most of the known housekeeping genes we examined, such as *B2M*, *PGK1*, *PPIA*, *RLP0*, *SDHA*, *TFRC*, *YWHAZ*, and others, exhibited similar expression levels in both our SZ and control samples. However, a smaller subset of recognized housekeeping genes (*ACTB*, *CYY1*, *EIF4A2*, *HPRT1*, and *HBMS*) showed significantly lower expression, while *TBP* and *GUSB* exhibited higher expression in the SZ samples (Table 4). These findings imply underlying biological distinctions between the two groups that extend beyond the genes potentially associated with SZ. These differences may arise from various other factors present in SZ subjects. Among the most prominent of these factors are inflammation, cellular stress, and alterations in cellular metabolism.

## 4. Discussion

We found that *NLRP1* mRNA expression is substantially higher in the DLPFC and MOFC of six SZ subjects compared to six controls. The immunohistochemical analysis of NLRP1 immunoreactivity in the MOFC from the right hemisphere of seven SZ subjects showed significantly higher numbers of NLRP1-expressing pyramidal cells in layers III, V, and VI compared to five controls, thus supporting our initial observation and suggesting NLRP1 inflammasome activation in SZ subjects. Unlike control samples where only a lower number of layer III pyramidal cells were positive, SZ samples had, on average, a higher number of layers III, V, and VI NLRP1-immunoreactive pyramidal cells. These results are novel, as previous methods and studies did not allow this kind of analysis or have not included the analysis of the NLRP1 inflammasome. Layer III pyramidal neuron dysfunction in SZ aligns with working memory deficits, while impairments of pyramidal neurons in layer V and particularly layer VI are consistent with the disruption of predictive processing. Layer IV was devoid of changes, showing very few, if any, NLRP1-positive cells. 

Inside the brain, the majority of immune actions are conducted by microglia, as they control innate immune responses and survey the brain parenchyma [68]. During brain development, microglia play essential roles by regulating cell and synapse numbers, eliminating excess immature synaptic connections, and producing mediators whose profiles vary based on the microglia’s activation state, consequently influencing different neurodevelopmental processes [69,70,71]. The mechanisms to explain the involvement of immune system, including microglia, in the pathogenesis of SZ include changes in the developing fetal brain due to prenatal infection and MIA [35,40,72,73,74,75]. For a recent, comprehensive overview of MIA, refer to [76], and for a potential mechanistic framework toward a deeper understanding of SZ immunophenotype, see [77]. As genes overexpressed in SZ brains were significantly enriched among MIA-induced differentially methylated genes in the fetal brain in a cell-type-specific manner (upregulated genes in layer V pyramidal neurons were highly significantly enriched among hypomethylated genes on gestational days 9 and 17), it is believed that MIA-driven methylation changes during gestation may influence SZ gene expression signatures in the adult brain [78]. MIA can also impact the epigenetic patterns of the genes involved in SZ onset [78]. Indeed, it has been shown that inflammation during early development can change the methylation of some genes and alter their expression in adulthood [78,79,80]. One way of activating inflammatory responses is the activation of the inflammasomes. A recent study has shown that MIA can cause SZ-like behavior in rats due to the activation of Toll-like receptors (TLR) and inflammasomes [40].

Our study indicates significant overexpression, i.e., an activation of the NLRP1 inflammasome in cells of the frontal lobe of individuals with SZ compared to controls. The overexpression of the NLRP1 inflammasome was most pronounced in pyramidal neurons in SZ brains. This led us to conclude that the activation of the “neuronal” NLRP1 inflammasome likely precedes selective atrophy, particularly decreased dendritic arborization and profound dendritic spine loss visualized using the Golgi stain, in deep layer III pyramidal neurons in the PFC in SZ. We hypothesize that this occurs after initial insults during the perinatal or early postnatal period. These initial changes, also known as “the first hit”, might lead to a vulnerability of the cortex to stressors during adolescence and early adulthood, eventually triggering dysfunctional immune pathways and apoptosis [81].

It remains to be examined whether the NLRP1 inflammasome is activated similarly to the NLRP3 inflammasome (as described by [82]). The NLRP3 inflammasome, composed of NLRP3, ASC (apoptosis-associated speck-like protein containing a caspase-activation and recruitment domain), and caspase-1, assembles inside microglia upon activation. This leads to the activation of the NF-κB signaling pathway, increased cleavage and activity of caspase-1, as well as downstream IL-1β and IL-18 release, activation of caspase-6, and cleavage of gasdermin D. To test the hypothesis of early NLRP1 inflammasome activation in SZ, a comprehensive analysis will be required, including appropriate model systems. Since an inflammasome can be activated and reactivated in neighboring cells due to extracellular ASC heteromer uptake, this creates a vicious cycle by promoting discontinued but constant microglial activation and severe inflammation that can spread within affected areas [8,83,84]. This may be one of the reasons why SZ might have a non-linear course characterized by a continuum of typical remissions and exacerbations in many patients.

Deficits in predictive processing are considered the key pathophysiological disturbance in SZ [16,17]. In individuals with SZ, the disruption of predictive processing impacts phenomenal consciousness (also called access consciousness or C0), giving rise to positive symptoms [85,86]. It also significantly affects self-reflection [87] and the experience of one’s body and the sense of “ownership” over it [88], thus undermining the metacognition and self-monitoring of their lived awareness (C1 consciousness), which in healthy individuals gives a subjective sense of error (reality check or C2 consciousness) [89]. Prediction errors update the current understanding of a situation through a search for meaning, with this learning signal strongly modulated by dopamine [90]. If, for any reason, the comparison of reality with internal representations performed through predictive processing by the PFC does not align (resulting in a reality check failure), symptoms of psychotic episodes or disorders may emerge—often within the SZ spectrum. However, a single psychotic episode or disorder can also be triggered, for example, by methamphetamines. Methamphetamines act as elicitors of paranoia in humans, a state associated with a stronger prior on volatility and elevated sensitivity to perceived changes in the task environment. Methamphetamine exposure in rats recapitulates this impaired, uncertainty-driven belief updating and rigid anticipation of a volatile environment [91]. In a mouse model, both chronic and acute methamphetamine treatment upregulated the expression of genes related to dopamine and serotonin metabolism in the striatum and PFC, suggesting a potential mechanism for how methamphetamine elicits an individual’s psychosis risk [92].

Long-term and heavy use of methamphetamine can increase the risk of developing psychotic symptoms, including hallucinations and delusions, which may resemble symptoms of SZ. Intriguingly, both NLRP1 and NLRP3 inflammasome overexpression, along with an increased induction of apoptosis and inflammation, were documented in the hippocampus by Western blotting, immunohistochemistry, and the terminal deoxynucleotidyl transferase dUTP nick end-labeling (TUNEL) method in eleven patients with methamphetamine use disorder [93]. Individuals with SZ also have an increased predilection for addiction, worsened by a loss of top-down control, contributing to more pronounced habitual tendencies and compulsive drug-seeking [94]. Compared to the general population, individuals with SZ are 4.6 times more likely to have a substance abuse diagnosis [95]. Here, it should be noted that this kind of cognitive control is distinct from general (fluid) intelligence, working memory, cognitive flexibility, planning, shifting attention, organizing thoughts, and problem-solving, mediated by networks of the DLPFC [96], and is more closely related to response inhibition and decision-making mediated by networks of the OFC. The DLPFC is especially hypoactive in persons with chronic SZ due to the selective atrophy of the deep layer III pyramidal neurons (for a review, see [81]). The transcranial direct current stimulation (tDCS) of the DLPFC has been shown to enhance working memory and suppress pathological γ power elevations in SZ subjects [97]. The MOFC integrates social and emotional information and its activity is concerned with monitoring, learning, and predicting the likely outcomes of actions related to the reward value of reinforcing stimuli, thus contributing to decision-making processes, especially subjective rewards [98], with more complex or abstract rewards such as monetary gain being represented more anteriorly than less complex ones such as taste, whereas lateral OFC activity is associated with negative reinforcing stimuli [99]. MOFC is considered a key component of the default mode network involved in self-referential thinking and understanding others’ thoughts, beliefs, intentions, and emotions (“theory of mind” abilities) [100,101]. 

The self-domestication theory suggests that humans have undergone a process of self-selection for traits such as reduced aggression, increased social tolerance, and cooperation [102,103]. Therefore, certain evolutionary pressures favored individuals who could live and work together more harmoniously, leading to the development of traits that are often associated with domesticated animals. One of three major hypotheses for self-domestication as an evolutionary root for SZ [102] considers the neoteny of synaptic spines of the association pyramidal neurons in the PFC [104]. In essence, the excessive generation and developmental remodeling of synaptic spines continue after adolescence under stronger dopaminergic innervation, possibly due to the human-specific expression of *TH* (tyrosine hydroxylase) gene in a subset of inhibitory neurons in the DLPFC [105] in the second half of the second and the third decade of life before complete stabilization in adult values. This has probably given humans unprecedented opportunity to reach the highest levels of intrinsic motivation and cognitive abilities, while burdening them with increased susceptibility to the development of abnormal neural circuits in adolescence and post-adolescence, manifested in neuropsychiatric disorders such as SZ.

Years of study have emphasized the significance of the optimal activation of cortical dopamine receptors in governing cognition associated with the PFC in humans [106,107]. The expansion of the neocortex in primate evolution has been paralleled by increased innervation by dopamine [108]. An analysis of axon length density to neuron density among species by Raghanti and collaborators revealed that humans and chimpanzees together deviated from macaques in having increased dopaminergic afferents in layers III and V/VI in the PFC [106]. Both the pyramidal and non-pyramidal cells of PFC, which include DLPFC and MOFC, express the D1-like (D1R and D5R) and D2-like (D2R, D3R, and D4 R) families of dopamine receptors, indicating fine regulation that is disturbed in SZ [109,110]. Individuals with SZ often face challenges in distinguishing between their own thoughts and external stimuli, leading to false beliefs or perceptions that are not in line with reality (delusions) and hallucinations (positive symptoms of SZ). These symptoms are associated with the increased dopamine activity of the mesocortical pathway through the nucleus accumbens, augmenting D2-like receptor activation. This effect is blocked by antipsychotics [111]. Besides cognitive deficits, other negative symptoms such as reduced motivation, reduced goal-directed behavior, anhedonia, thought disorder, poverty of speech (alogia), and social withdrawal have been linked to DLPFC dysfunction due to reduced D1R activation [111]. Since cognitive and negative symptoms of SZ are less directly associated with the dysfunction of D2-like receptors, they are more difficult to treat with antipsychotics, especially because some patients may lack insight into the extent and impact of their symptoms [112,113].

## 5. Limitations of the Study and Conclusions

These initial results showed significantly increased NLRP1 inflammasome activation in the dorsolateral prefrontal and medial orbitofrontal cortex in SZ compared to control brains, which will require detailed analyses of NLRP1 and other inflammasome-related proteins in additional brain regions. Follow-up on new information can be found at: https://www.proteinatlas.org/ENSG00000091592-NLRP1/brain (accessed on 22 February 2024). The primary limitations of this study include a small sample size for *NLRP1* expression analysis and immunohistochemical analysis performed only in MOFC brain tissue for the right hemisphere. While elevated levels of *NLRP1* mRNA and protein expression can indicate activated inflammasomes, it does not automatically confirm their activation, and it is not the only indicator. NLRP1 is indeed a crucial component of the neuronal inflammasome, but other factors, such as the presence of activating stimuli, the availability of accessory inflammasome components, and the overall cell state, can also play a role in inflammasome activation. That being said, the increase in *NLRP1* mRNA and NLRP1 protein expression may indicate a cellular response to various potential threats or stressors. Although inflammasomes are typically activated by certain bacteria, viruses, or pathogens, they can also respond to other stimuli like stress, trauma, or certain drugs. Some published hypotheses regarding the activation of the NLRP1 inflammasome include its activation by various viral proteases [114], possibly during intrauterine development in the case of SZ. Predisposition by certain *NLRP1* gene polymorphisms [115] and the prior activation of the NLRP3 inflammasome in microglia, e.g., by extracellular amyloid [82] or templated tau seeds [116], are also suggested, with conceivable spreading by ASC proteins (“specks”) and possibly intracellular amyloid or other intracellularly generated amyloid precursor protein-generated metabolites and other harmful molecules.

Another challenge in interpreting our results is that inflammasome activation involves several steps beyond increased *NLRP1* expression. These steps include its assembly into the inflammasome complex, the recruitment of other proteins, and the subsequent cleavage and activation of proinflammatory cytokines. When an inflammasome is activated, it cleaves pro-inflammatory cytokines, such as IL-1β or IL-18, from their inactive precursor forms. These cytokines then can act on a variety of cells to promote the inflammatory response. It should be kept in mind that our analysis is limited to the dorsolateral prefrontal cortex and the medial orbital cortex. Therefore, confirming inflammasome activation requires further investigations, such as assessing the formation of the NLRP1 inflammasome complex and measuring caspase-1 activation and cytokine release. Upstream and downstream alterations due to elevated NLRP1 and associated signaling pathways should also be investigated, as well as inflammatory responses and changes related to neuroinflammation, synaptic dysfunction triggered by inflammation, and neuronal pyroptosis. Despite the multi-faceted nature of inflammasomes, the heterogeneity of pathophysiological alterations, and the consequent variability in clinical presentation, along with all the mentioned limitations of this preliminary research, our results, including the differences in the housekeeping gene expression between the SZ and CON groups, strongly suggest that it is worthwhile to further investigate the role and significance of inflammasome activation in SZ.

Ultimately, all comparative evaluation studies of efficacy and safety to date have supported the use of anti-inflammatory adjuvant therapy over antipsychotics alone. However, despite the recognition of inflammation in individuals affected with SZ, this important discovery and overall significant positive effects of various anti-inflammatory agents (such as acetylsalicylic acid, celecoxib, omega-3 fatty acids, estrogen, selective estrogen receptor modulator, pregnenolone, *N*-acetylcysteine, minocycline, davunetide, and erythropoietin) in reducing total, positive, and negative symptoms scores in the PANSS, as well as significant cognitive improvements with minocycline and pregnenolone augmentation therapy without significant differences in side effects compared with placebo (for a review, see [117]), have not yet resulted in expected new treatments. The limited success of clinical trials using anti-inflammatory drugs likely stems from the inability to pinpoint the specific inflammatory mechanisms targeted by existing medications. Perhaps, not all patients show initial inflammation. In other words, certain individuals are more prone to heightened inflammation and are also responsive to such treatment (for a review, see [32]). This might also be part of the explanation as to how, despite the substantial heritability of SZ (h^2^ = 65–79%) [118], the identified risk variants collectively contribute to only a very limited portion (h^2^_SNP_ = 24%) of the total variability in the susceptibility of the phenotype [119]. In fact, in a longitudinal study on 84 patients with a clinical diagnosis of schizophrenic disorders (ICD-10 “F2x.x”), a multi-layer neural network model based on the backpropagation supervised learning algorithm identified a subgroup of 22.5% of patients with SZ who exhibited a significant correlation between global SZ scores and immunoglobulin M (IgM) levels, along with a correct prediction of the response to therapy in 94.4% of them [120]. Once again, non-steroidal and anti-inflammatory drugs, including acetylsalicylic acid, and COX-2 inhibitors showed significant positive effects as adjunctive treatments in SZ.

In conclusion, further research is needed to determine the neuroinflammation profile in individuals with SZ, involving comprehensive data collection, the molecular analysis of peripheral and central inflammatory biomarkers, and the consideration of the role of the NLRP1 inflammasome as a potential prognostic biomarker and therapeutic target, with a focus on patients with homogeneous clinical profiles for personalized insights into anti-inflammatory treatment effects. Additional specificity in future studies could be made by performing double labeling analyses using specific markers of pyramidal neurons, such as RBP4 and EMX1.

## Figures and Tables

**Table 1:** Demographic data of subjects with schizophrenia and control subjects.

Case	Sex (F/M)	Age (Years)	Cause of Death
Subjects with schizophrenia (SZ)			
SZ1	F	42	Drug poisoning
SZ2	F	47	Sudden cardiac arrest
SZ3	M	50	Heart failure
SZ4	F	50	Not known (no autopsy conducted)
SZ5	F	56	Aortic dissection
SZ6	F	57	Sudden cardiac arrest
SZ7	F	58	Suicide by jumping from a height
SZ8	M	59	Suicide by hanging
SZ9	M	59	Suicide by hanging
SZ10	M	81	Not known (no autopsy conducted)
Mean ± SD		55.9 ± 10.5	
Control subjects (CON)			
CON1	F	40	Heart failure
CON2	M	42	Heart failure
CON3	M	54	Heart failure
CON4	M	55	Heart failure
CON5	F	60	Sudden cardiac arrest
CON6	F	61	Heart failure
CON7	M	63	Sudden cardiac arrest
CON8	M	64	Sudden cardiac arrest
CON9	M	66	Sudden cardiac arrest
CON10	M	73	Pulmonary embolism
Mean ± SD		57.8 ± 10.4	

CON, control subjects; F, female; M, male; SD, standard deviation; SZ, schizophrenia subjects.

**Table 2:** Tissue characteristics of the control (CON) and schizophrenia (SZ) brain samples analyzed for the expression of the *NLRP1* gene (dataset line 18,408 in Supplementary Table S1). RIN was calculated using a proprietary algorithm of Agilent Technologies, Santa Clara, CA, USA. All *p*-values of the Shapiro–Wilk test greater than α = 0.05 mean that a sample comes from a normally distributed population.

CON Subject/RIN	Brain Tissue Block	*NLRP1* mRNA exp. in LH DLPFC	*NLRP1* mRNA exp. in RH DLPFC	*NLRP1* mRNA exp. in LH MOFC	*NLRP1* mRNA exp. in RH MOFC	SZ Subject/RIN	Brain Tissue Block	*NLRP1* mRNA exp. in LH DLPFC	*NLRP1* mRNA exp. in RH DLPFC	*NLRP1* mRNA exp. in LH MOFC	*NLRP1* mRNA exp. in RH MOFC
CON3 7.5	LH DLPFC	11.7				SZ8 8.7	RH DLPFC		13.2		
	RH DLPFC		14.2				LH DLPFC	12.4			
	LH MOFC			14.1			RH MOFC				12.9
	RH MOFC				11.3		LH MOFC			13.6	
CON5 6.9	LH DLPFC	13.2				SZ5 7.4	RH DLPFC		13.6		
	LH MOFC			11.7			LH DLPFC	13.0			
	RH DLPFC		13.1				RH MOFC				16.0
	RH MOFC				12.4		LH MOFC			16.0	
CON6 8.6	RH DLPFC		13.2			SZ6 5.0	RH DLPFC		14.6		
	RH MOFC				11.7		LH DLPFC	13.7			
	LH DLPFC	13.1					RH MOFC				14.0
	LH MOFC			12.4			LH MOFC			12.4	
CON1 7.7	RH DLPFC		14.1			SZ3 8.1	RH DLPFC		13.2		
	LH DLPFC	12.2					LH DLPFC	12.3			
	RH MOFC				13.0		RH MOFC				16.3
	LH MOFC			11.6			LH MOFC			16.5	
CON4 8.6	RH DLPFC		12.5			SZ2 8.0	RH DLPFC		17.2		
	LH DLPFC	16.4					LH DLPFC	16.6			
	RH MOFC				15.0		RH MOFC				15.0
	LH MOFC			14.0			LH MOFC			15.7	
CON2 8.8	RH DLPFC		12.4			SZ1 5.0	RH DLPFC		15.7		
	LH DLPFC	15.5					LH DLPFC	13.8			
	RH MOFC				16.7		RH MOFC				19.1
	LH MOFC			15.6			LH MOFC			14.3	
	Shapiro–Wilk *p*	0.44	0.36	0.48	0.47		Shapiro–Wilk *p*	0.12	0.29	0.76	0.96
8.0	Mean	13.67	13.26	13.25	**13.99**	7.0	Mean	13.63	14.57	14.75	**15.53**
0.7	SD	1.87	0.85	1.58	2.32	1.7	SD	1.60	1.58	1.58	2.14
	Total mean	**13.543** (N = 24)					Total mean	**14.620** (N = 24)			
	Total SD	1.7391					Total SD	1.7415			
		Unpaired two-tailed Student’s *t*-test: T = −2.1498, d.f. = 46, ***p* = 0.03687** Non-parametric two-sample, two-tailed Mann–Whitney U test: Z = −2.4436, ***p* = 0.01454**									

CON, control; DLPFC, dorsolateral prefrontal cortex (Brodmann’s area 46); exp., expression; LH, left hemisphere; MOFC, medial orbitofrontal cortex (Brodmann’s area 11/12); RH, right hemisphere; RIN, RNA integrity number; SD, standard deviation; SZ, schizophrenia.

**Table 3:** Quantitative assessment of the number of NLRP1-expressing pyramidal neurons in the MOFC of control (CON) and schizophrenia (SZ) subjects. MOFC, medial orbitofrontal cortex (Brodmann’s area 11/12); RH, right hemisphere; SD, standard deviation.

CON Subject	Number of NLRP1-Positive Pyramidal Neurons in RH MOFC	Area [mm^2^]	Average Density/mm^2^	SZ Subject	Number of NLRP1-Positive Pyramidal Neurons in RH MOFC	Area [mm^2^]	Average Density/mm^2^
CON8	254	47.8	5.3	SZ5	1248	93.6	13.3
CON9	1020	184.1	5.5	SZ8	1224	87.8	13.9
CON10	558	58.0	9.6	SZ7	830	62.3	13.3
CONT5	422	89.7	4.7	SZ9	648	35.8	18.1
CON7	1064	114.7	9.3	SZ10	1602	158.7	10.1
				SZ6	1037	64.4	16.1
				SZ4	1010	83.3	12.1
Mean	663.6	98.86	**6.88** (N = 5)	Mean	1085.6	83.7	**13.84** (N = 7)
SD	362.16	54.49	2.37	SD	310.13	38.44	2.61
Unpaired two-tailed Student’s *t*-test: T = −4.8068, d.f. = 10, ***p* = 0.00088** Non-parametric two-sample, two-tailed Mann–Whitney U test: Z = −2.7656, ***p* = 0.005681**							

**Table 4:** Comparison of housekeeping gene expression between schizophrenia (SZ) and control (CON) samples. Next to the name of each gene in the second column is its Entrez Gene ID number and the dataset line of Supplementary Table S1 in which the mRNA expression of that gene for all samples can be found.

Gene	Entrez Gene ID/Dataset Line	Average Expression in 24 Samples of Six CON Subjects ± SD		Average Expression in 24 Samples of Six SZ Subjects ± SD	Difference
*ACTB*	3036924 8960	6466.17 ± 1028.01	>	5356.90 ± 1821.50	**Significant** (T = 2.60, d.f. = 46, ***p* = 0.013**)
*B2M*	3592023 13433	336.17 ± 162.40	>	261.70 ± 152.95	Non-significant (T = 1.64, d.f. = 46, *p* = 0.109)
*CYC1*	3120051 6681	155.984 ± 73.52	>	116.69 ± 28.53	**Significant** (T = 2.44, d.f. = 46, ***p* = 0.019**)
*EIF4A2*	2656738 8617	1648.94 ± 583.95	>	1265.58 ± 380.49	**Significant** (T = 2.69, d.f. = 46, ***p* = 0.010**)
*GUSB*	3053691 951	33.98 ± 5.69	<	37.50 ± 6.37	**Significant** (T = −2.02, d.f. = 46, ***p* = 0.049**)
*HMBS*	3351841 17437	23.46 ± 4.81	>	18.20 ± 3.69	**Significant** (T = 4.25, d.f. = 46, ***p* = 0.0001**)
*HPRT1*	3991698 84	520.55 ± 251.05	>	354.93 ± 188.48	**Significant** (T = 2.58, d.f. = 46, ***p* = 0.013**)
*PGK1*	3982462 14144	3439.35 ± 615.11	>	3352.13 ± 577.90	Non-significant (T = 0.51, d.f. = 46, *p* = 0.506)
*PPIA*	3000073 19231	20.97 ± 7.28	<	21.84 ± 5.50	Non-significant (T = −0.47, d.f. = 46, *p* = 0.642)
*RPLP0*	3474344 6859	74.18 ± 14.20	<	80.61 ± 20.16	Non-significant (T = −1.28, d.f. = 46, *p* = 0.208)
*SDHA*	2798538 14738	1249.97 ± 172.91	>	1140.73 ± 296.17	Non-significant (T = 1.56, d.f. = 46, *p* = 0.126)
*TBP*	2937984 9928	102.00 ± 25.81	>	118.95 ± 25.85	**Significant** (T = −2.27, d.f. = 46, ***p* = 0.028**)
*TFRC*	2712632 5038	141.99 ± 35.76	>	134.22 ± 45.06	Non-significant (T = 0.66, d.f. = 46, *p* = 0.51)
*YWHAZ*	3146898 9013	1307.58 ± 299.20	<	1315.23 ± 334.26	Non-significant (T = −0.08, d.f. = 46, *p* = 0.934)

## Data Availability

Data are contained within the article and Supplementary Table S1. All data generated or analyzed during this study are available from the corresponding author upon reasonable request.

## Supplementary Materials

The following supporting information can be downloaded at: https://www.mdpi.com/article/10.3390/biom14030302/s1, Table S1: Gene Expression Raw Data.xls.
